# Pharmacokinetics of fluticasone furoate, umeclidinium, and vilanterol as a triple therapy in healthy volunteers

**DOI:** 10.5414/CP202390

**Published:** 2015-07-31

**Authors:** Noushin Brealey, Ashutosh Gupta, Jessica Renaux, Rashmi Mehta, Ann Allen, Alex Henderson

**Affiliations:** 1 Respiratory Medicines Development Centre, GSK, Uxbridge, UK,; 2 Quantitative Sciences India, GSK, Bangalore, India,; 3 Respiratory Clinical Pharmacology Science and Study Operations, GSK, Uxbridge, UK,; 4 Research Triangle Park, NC, USA,; 5 Clinical Pharmacology Modelling and Simulation Department, GSK, Stevenage, and; 6 Clinical Pharmacology Science and Study Operations, GSK, Stevenage, UK

**Keywords:** inhaled corticosteroid, long-acting muscarinic antagonist, long-acting β2 agonist, triple therapy, COPD

## Abstract

Objective: Two single-center, four-way, single-dose, crossover studies assessed the systemic exposure, systemic pharmacodynamics (PD), and safety profile of the closed triple fluticasone furoate/umeclidinium/vilanterol (FF/UMEC/VI) therapy compared with dual therapies. These are the first studies where pharmacokinetic (PK) profile assessment was possible for this inhaled triple fixed-dose combination product. Methods: Healthy volunteers were randomized to receive 4 consecutive inhalations (each administered as a single dose) via a single ELLIPTA^®^ dry powder inhaler: in study 1 (CTT116415/NCT01691547), FF/UMEC/VI at total doses of 400/500/100 μg, FF/UMEC 400/500 μg, UMEC/VI 500/100 μg, or FF/VI 400/100 μg; in study 2 (200587/NCT01894386), FF/UMEC/VI at total doses of 400/500/100 μg or 400/250/100 μg, FF/VI 400/100 μg, or UMEC/VI 250/100 μg. PK and PD parameters and safety were assessed. Results: Of 88 subjects, 95% completed both studies and received all planned treatments. Total systemic exposure was similar for FF, UMEC, and VI when administered as a triple therapy compared with FF/VI and UMEC/VI. No clinically significant systemic PD findings were detected. The incidence of adverse events was low and similar across treatment arms. Conclusions: Systemic exposure to all three components of the closed triple therapy, following single-dose delivery, was similar to that seen with the dual therapies FF/VI and UMEC/VI. The delivered lung dose and safety profile of all three agents, delivered via a single inhaler, are expected to be similar to those of the dual therapies.

## Introduction 

Patients with chronic obstructive pulmonary disease (COPD) experience increasing airflow obstruction and enhanced chronic inflammation within the respiratory system. Current guidelines recommend an incremental approach to treatment involving the combination of different drug classes as COPD severity progresses [1]. 

Triple combination therapy is increasingly used in COPD management [2, 3]. An inhaled corticosteroid (ICS)/long-acting muscarinic receptor antagonist (LAMA)/long-acting β_2_-adrenergic receptor agonist (LABA) combination is now a recommended second option for symptomatic COPD patients at high risk of exacerbations (GOLD grade D) [1]. Triple therapy, when administered as a once-daily single-dose combination from one inhaler, offers improved convenience of administration and a reduced burden of polypharmacy for patients with the potential for improved compliance compared to individual agents from separate inhalers. 

Fluticasone furoate/umeclidinium/vilanterol (FF/UMEC/VI), also known as the Closed Triple, is a once-daily ICS/LAMA/LABA therapy in phase III development in patients with COPD, for administration via the single-step activation ELLIPTA^®^ (ELLIPTA^®^ is a trade mark of the GSK group of companies) dry powder inhaler (DPI). Fixed-dose combinations of FF/VI and UMEC/VI, delivered via the ELLIPTA DPI, are approved in the United States (US), European Union, and Canada, among other countries/regions, for the treatment of moderate-severe COPD. 

Here, we present findings from the first two single-dose, healthy volunteer studies to assess pharmacokinetics (PK) of FF, UMEC, and VI administered as a closed triple therapy in a single inhaler compared with the same components administered as dual combinations. 

## Methods 

### Subjects

For both studies, eligible subjects were healthy nonsmokers aged 18 – 65 years. Key inclusion criteria were: body mass index 19 – 33 kg/m^2^ (study 1) or 18 – 33 kg/m^2^ (study 2); average QTcF < 450 msec; and a normal lung function of ≥ 80% forced expiratory volume in 1 second (FEV_1_) predicted and FEV_1_/forced vital capacity ≥ 0.7. All subjects provided written informed consent. 

### Study design

Study 1 (GSK/clinicaltrials.gov numbers: CTT116415/NCT01691547) and study 2 (200587/NCT01894386) were conducted in the same US center from December 17, 2012 to March 8, 2013, and July 15 to September 26, 2013, respectively. 

Both studies were randomized, single-dose, crossover trials; study 1 was double blind, and study 2 was open-label. A randomization schedule was generated using a validated computer system (RandAll; GSK, Brentford, UK), and subjects were subsequently assigned to receive 1 of 4 treatment sequences in random order. In study 1, subjects were randomized to receive 4 consecutive inhalations of each of the following treatments: FF/UMEC/VI, UMEC/VI, FF/VI, and FF/UMEC, containing doses of 100 μg FF, 125 μg UMEC, and 25 μg VI (total single doses: 400 μg FF, 500 μg UMEC, and 100 μg VI). Likewise, in study 2, subjects were randomized to receive 4 consecutive inhalations of each of the following treatments: FF/UMEC/VI, FF/VI, and UMEC/VI, containing doses of 100 μg FF, 125 μg (high dose) or 62.5 μg (low dose) UMEC, and 25 μg VI (total single doses: 400 μg FF, 500 μg (high dose) or 250 μg (low dose) UMEC, and 100 μg VI). In each study, all treatments were administered via the two-strip ELLIPTA DPI, with a 7 – 21-day washout period between treatments; UMEC/VI was administered as a blended combination in 1 strip, and FF was coadministered in the second strip. 

For both studies, the primary objective was to assess the systemic exposure of FF, UMEC, and VI following single inhaled doses (4 inhalations) of FF/UMEC/VI, UMEC/VI, or FF/VI, including (in study 2 only) the assessment of 2 UMEC dose levels (500 and 250 μg). Secondary objectives assessed systemic exposure of FF/UMEC (study 1 only), systemic pharmacodynamics (PD; study 1 only), and urine PK of UMEC (study 1 only). 

### Outcomes

****Pharmacokinetic analysis****

In study 1, plasma PK samples were collected predose and at 5, 15, and 30 minutes and 1, 2, 4, 6, 8, 12, 16, 24, 36, and 48 hours postdose, and urine PK samples were taken from 0 to 6, 6 to 10, 10 to 14, 14 to 18, and 18 to 24 hours. Urine was collected in study 1 only. To better characterize the concentration-time profile of VI within the first 15 minutes postdose in study 2, additional plasma PK samples were collected at 3, 7, 10, 12, and 45 minutes and 1.5 hours postdose. Samples were not collected at 16, 36, or 48 hours postdose in study 2. Plasma PK samples were obtained from 5 to 6 mL blood samples. Analysis of plasma and urine samples was performed by York Bioanalytical Solutions (managed by Bioanalytical Science and Toxicokinetics, Drug Metabolism and Pharmacokinetics, GSK). Analysis of FF, UMEC, and VI levels was conducted using validated analytical techniques (online supplement) with 10 pg/mL as the lower limit of quantification for each compound. 

PK parameters were calculated by standard noncompartmental analysis according to current working practice and using WinNonlin Professional 6.3 (Pharsight Corporation, Mountain View, CA, USA). The PK parameters and plasma concentrations estimated for each treatment group included: area under the plasma concentration-time curve (AUC_(0–t)_, AUC_(0–24)_, AUC_(0–t)_, (study 1: AUC_(0–8)_ for FF, AUC_(0–4)_ for UMEC, AUC_(0–2)_ for VI; study 2: AUC_(0–4)_ for FF, AUC_(0–2)_ for UMEC, AUC_(0–6)_ for VI)), time of last measurable concentration, terminal phase half-life, maximum observed plasma concentration (C_max_), and time to C_max_ (t_max_). 

****Pharmacodynamic analysis (study 1 only)****

Electrocardiograms (ECGs) were assessed from screening and predose on the 1^st ^day of each treatment period, then at 5, 15, and 30 minutes and 1, 2, 4, 12, and 24 hours postdose. Heart rate was assessed from 0 to 4 hours, and blood samples to assess systemic PD of VI (serum potassium, and blood glucose) were also collected predose and at 30 minutes, 1, 2, and 4 hours postdose, then assayed using approved analytical methodology (Supplementary material). 

****Safety analysis****

Adverse events (AEs), serious AEs (SAEs), laboratory values, vital signs, ECGs, and physical examinations were monitored for safety assessment. AEs were coded using the Medical Dictionary for Regulatory Activities. 

****Statistical analysis****

In study 1, the primary comparisons were FF/UMEC/VI vs. FF/VI, and FF/UMEC/VI vs. UMEC/VI. A secondary comparison was FF/UMEC/VI vs. FF/UMEC. Posthoc analyses of individual concentration-time profiles for VI were also performed. The comparisons in study 2 were FF/UMEC(500)/VI vs. FF/UMEC(250)/VI, FF/UMEC(250)/VI vs. FF/VI, and FF/UMEC(250)/VI vs. UMEC(250)/VI. 

No formal hypothesis testing was conducted for either study as an estimation approach was adopted. Data were reported using statistical analysis software (SAS, Cary, NC, USA) version 9.1.3. on a UNIX platform. Log_e_-transformed AUC_(0–t)_, and C_max_ were analyzed separately using a mixed-effects model, with period and treatment fitted as fixed effects and subject as a random effect. For comparison of the 2 UMEC dose levels, UMEC PK parameters were dose normalized prior to analysis by dividing the respective PK parameter values by the received planned dose. For each comparison of interest, point estimates and corresponding 90% confidence intervals (CIs) were calculated for the ratio (for PK) or difference (for PD) between the means of the test and reference treatments. Although the studies were not conducted as formal bioequivalence studies, treatments were considered comparable if the 90% CIs of the ratio between the triple and dual therapies were within 0.8 – 1.25 (standard bioequivalence acceptance limits). Where small differences were seen, these were further assessed for their clinical relevance. Each derived PD endpoint was analyzed using a mixed effects model. 

Planned enrollment for each study was 44 subjects, with the target of ≥ 36 subjects completing dosing and critical assessments. See Supplemental material for sample size assumptions. 

All subjects who received ≥ 1 dose of study medication were included in the “all subjects” population and used for the PD and safety data. The “PK population” was defined as subjects within the “all subjects” population for whom a PK sample was obtained and analyzed. 

****Posthoc exploratory analyses****

Three posthoc exploratory analyses were performed. The first two analyses, described below, investigated the hypothesis that VI PK may not have been optimally defined in study 1, due to a low number of time points in the sampling schedule. The 3^rd^ analysis, presented in the Supplemental material, was undertaken to support future development of VI as a monotherapy. 

*Population* *pharmacokinetic* *modeling* *(study 1)*

Posthoc population PK modeling of log-transformed VI concentration-time data was performed using a three-compartment population PK model to investigate the impact of the PK sampling schedule on VI C_max_. Based on a previous VI population PK modeling analysis (data on file), additional time points were included at 1 minute intervals between 2 and 14 minutes to improve C_max_ characterization. Individual AUC_(0–24h)_ was derived as the ratio of the nominal dose divided by the individual posthoc estimate of apparent clearance from the final population PK model. Individual C_max_ was derived from the predicted concentration-time profile obtained from the model for each subject. 

*Exploratory* *pharmacokinetic* *analysis* *(study 2)*

Posthoc exploratory PK analysis was performed to repeat the PK analysis from study 2, with the following PK time points removed: 3, 7, 10, 12, 45, and 90 minutes. In addition, FF and VI PK parameters were compared between studies 1 and 2 to assess FF/UMEC(500)/VI with FF/VI. PK parameters were analyzed using a mixed-effects model, as described in the original analysis. 

## Results 

### Subject disposition and demographic characteristics (Table 1) 

In study 1, 44 subjects were randomized and included in the “all subjects” population: 93% (n = 41) completed the study and received all planned treatments; three subjects discontinued study medication due to an SAE (n = 1) or investigator discretion (n = 2). The PK population comprised 98% of subjects (n = 43). 

In study 2, 44 subjects were randomized and included in the “all subjects” population: 98% (n = 43) completed the study; 1 subject withdrew due to a protocol violation. All subjects were included in the PK population. 

The demographic characteristics were similar for each study. 

### Pharmacokinetics

PK parameters are reported for FF (Table 2a, Table 2b), UMEC (Table 3a, Table 3b), and VI (Table 4a, Table 4b). Table 5a and Table 5b provide the statistical comparisons of systemic exposure between the triple and dual therapies. 

****Fluticasone furoate****

**Figure 1.**
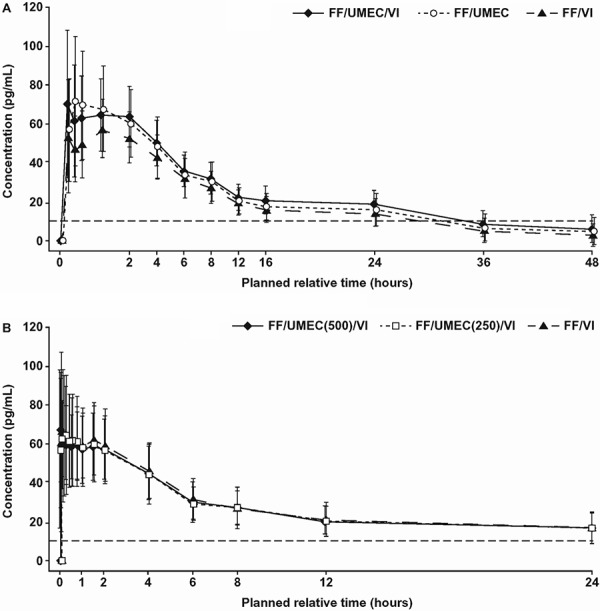
FF PK profile in (a) study 1 (CTT116415)^a^ and (b) study 2 (200587)^b^. The horizontal dashed line indicates the lower limit of quantification. ^a^Total dose as 4 consecutive doses: 400 μg FF, 500 μg UMEC, and 100 μg VI. ^b^Total dose as 4 consecutive doses: 400 μg FF, 500 μg or 250 μg UMEC, and 100 μg VI.

FF was quantifiable from 3 to 12 minutes postdose, dependent on therapy and study, and in the majority of subjects ≤ 24 hours postdose (Figure 1A, B). 

In both studies, similar FF systemic exposure (AUC and C_max_) was observed between triple therapy and the FF/VI and FF/UMEC dual therapies, except for a slightly higher C_max_ in study 1 where the triple vs. FF/VI ratio was 1.23 (90% CI, 1.14 – 1.33; Table 5a). This difference was considered to be clinically nonrelevant. In study 2, there were no observed differences in FF systemic exposure (C_max_ and AUC) between the two triple combinations (500 and 250 μg UMEC). 

****Umeclidinium****

**Figure 2.**
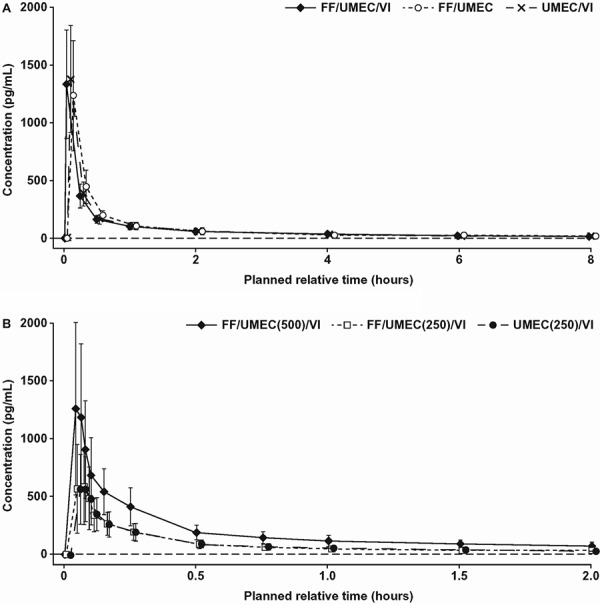
UMEC PK profile in (a) study 1 (CTT116415)^a^ and (b) study 2 (200587)^b^. The horizontal dashed line indicates the lower limit of quantification. ^a^Total dose as 4 consecutive doses: 400 μg FF, 500 μg UMEC, and 100 μg VI. ^b^Total dose as 4 consecutive doses: 400 μg FF, 500 μg or 250 μg UMEC, and 100 μg VI.

UMEC was quantifiable for all treatments from 5 minutes to 24 hours (study 1) and 3 minutes to 6 hours (study 2) postdose in the majority of subjects (Figure 2A, B). At 24 hours postdose in study 2, UMEC was quantifiable in 55% of subjects following FF/UMEC(500)/VI compared with only 14% and 12% subjects following FF/UMEC(250)/VI and UMEC/VI, respectively. 

In study 1, similar UMEC systemic exposure (AUC and C_max_) was observed between triple therapy and the UMEC/VI and FF/UMEC dual therapies. UMEC urine PK parameters, such as the percent of dose excreted in urine over 24 hours (~2%), urinary half-life (13 – 16 hours), and renal clearance (12 L/h), were also similar between the triple and both dual therapies. In study 2, UMEC systemic exposure (C_max_ and AUC) increased in proportion with increasing UMEC dose and was similar between triple and dual therapies for all parameters. 

****Vilanterol****

VI was quantifiable at 5 minutes (study 1) and 3 minutes (study 2) postdose, and very few subjects showed detectable VI at 24 hours postdose following triple, FF/VI, and UMEC/VI therapies. Similar VI systemic exposures, in terms of AUC, were observed between the triple and FF/VI or UMEC/VI therapies (Table 5a). C_max_ was slightly higher for VI when administered as the triple therapy vs. FF/VI (1.46 [90% CI, 1.38 – 1.56]) and as the triple therapy vs. UMEC/VI (1.33 [90% CI, 1.25 – 1.42]), but this was not considered to be clinically significant (Table 5a). 

**Figure 3.**
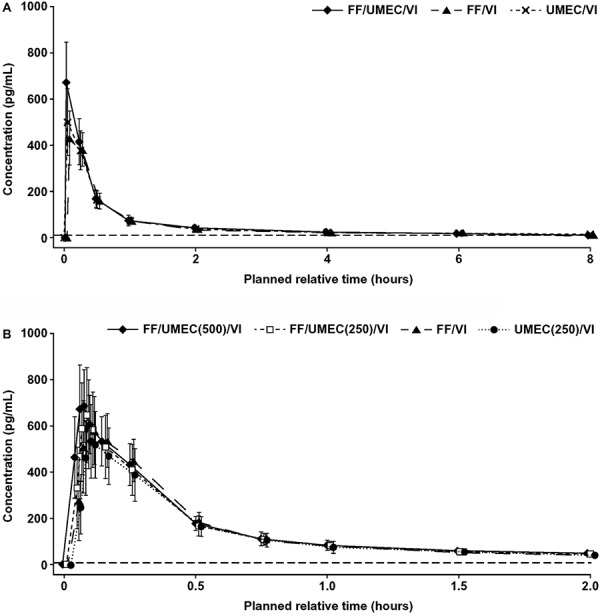
VI PK profile in (a) study 1 (CTT116415)^a^ and (b) study 2 (200587)^b^. The horizontal dashed line indicates the lower limit of quantification. ^a^Total dose as 4 consecutive doses: 400 μg FF, 500 μg UMEC, and 100 μg VI. ^b^Total dose as 4 consecutive doses: 400 μg FF, 500 μg or 250 μg UMEC, and 100 μg VI.

In study 2, VI systemic exposure (AUC and C_max_) was similar between the triple and FF/VI (Figure 3A), and between the two triple therapies (500 and 250 μg UMEC; Figure 3B). VI systemic exposure was also similar in terms of AUC between the triple and UMEC/VI; however, C_max_ was slightly higher following the triple therapy compared to UMEC/VI (1.21 [90% CI, 1.14 – 1.27]; Table 5b). This difference was not considered to be clinically significant. 

### Pharmacodynamics (study 1 only; Table 6)

There were no differences in heart rate for FF/UMEC/VI compared with either UMEC/VI or FF/VI. Maximum and weighted mean change (0 – 4 hours) from baseline heart rate increased for FF/UMEC/VI vs. FF/UMEC (mean difference ~ 14 and ~ 6 bpm, respectively; data not shown). These differences were not considered to be clinically significant. No clinically relevant difference in weighted mean change from baseline potassium level was observed for any triple vs. dual comparison. Weighted mean changes from baseline in blood glucose levels did not differ for FF/UMEC/VI vs. either UMEC/VI or FF/VI. A small difference in weighted mean change from baseline in blood glucose was reported with FF/UMEC compared with the triple therapy (~ 0.5 and 0.15 mmol/L, respectively; data not shown). These small increases, which are not considered to be clinically significant, can be attributed to the VI component and are similar to those seen in previous studies with FF/VI (800/100 μg) [4]. 

### Safety

Few AEs were reported (Table 7), and none were deemed by the investigator to be related to study treatment. No notable differences in AE incidence were observed between treatment groups. One SAE of diabetes mellitus was reported (study 1), which, upon further investigation, was established to be a previously undiagnosed case of type 2 diabetes mellitus. 

### Posthoc exploratory analyses

****PK population pharmacokinetic modeling (study 1; Supplementary Table 1)****

Posthoc PK data were modeled with fixed input times ranging from 2 to 12 minutes. Mean VI C_max_ was estimated to range from 620 to 690 pg/mL, with maximum C_max_ values observed for input times of 7 – 9 minutes. 

****Exploratory pharmacokinetic analysis (study 2; Supplementary Table 2)****

In the posthoc exploratory analysis of study 2 PK data, there was a greater difference in VI C_max_ for the triple vs. dual therapies when the additional time points were removed, compared with the full study 2 data. 

## Discussion 

These were the first studies to assess the PK of a new, closed, triple (FF/UMEC/VI) fixed-dose combination therapy vs. the dual therapies of FF/VI, UMEC/VI, and FF/UMEC in healthy volunteers. Overall results from the two studies suggest no clinically relevant differences in systemic exposure of FF, UMEC, or VI when administered as a triple vs. dual therapy. 

In study 1, an increase in FF C_max_ was reported with the triple therapy vs. FF/VI. This may reflect the small differences in absorption kinetics and be due to the t_max_ difference for FF between the triple therapy (0.23 hours) and FF/VI (1.0 hours). No difference in FF systemic exposure was observed in study 2 when FF was administered in the triple therapy with 2 different UMEC doses. 

VI C_max_ was slightly higher in study 1 following triple therapy compared with either FF/VI or UMEC/VI. Assessment of individual VI time profiles in study 1 suggested that C_max_ may have occurred earlier for the triple combination (~ 5 minutes), whereas C_max_ for FF/VI and UMEC/VI may have occurred later, between the 5 and 15-minute time points. Hence, the C_max_ value recorded for VI administered as a dual therapy may represent an underestimate in study 1. It is therefore possible that the small differences seen between the VI C_max_ of the triple combination and FF/VI and UMEC/VI may represent an apparent increase in the ratios of the respective VI C_max_ comparisons. 

The VI PK findings from both studies were therefore investigated in two posthoc exploratory analyses. The outcome of these concluded that the PK differences for VI C_max_ were most likely influenced by the low number of time points ~ 7 – 9 minutes in the sampling schedule. Additional time points were therefore included in study 2 to ensure more accurate characterization of C_max_. There was no observed difference in VI systemic exposure (C_max_ and AUC) in study 2 between FF/UMEC/VI and FF/VI; however, VI C_max_ following triple therapy was slightly higher compared with UMEC(250)/VI. This increase was less pronounced than that observed in study 1, and the difference is not considered clinically significant as the overall systemic exposure (in terms of AUC) was similar between the triple and dual UMEC/VI therapies. 

Where differences in C_max_ were observed for VI, these were not associated with any clinically relevant differences in the corresponding pharmacologically-related PD endpoints. This is despite the doses in this study being 4 times the proposed therapeutic dose, which represents a twelve-fold higher systemic exposure in healthy subjects than seen in patients with COPD (BREO^®^ ELLIPTA^®^ Prescribing Information, GSK [5]). 

## Conclusion 

Overall results from the two studies suggest no clinically relevant differences in systemic exposure of FF, UMEC, or VI when administered as a triple therapy vs. the approved FF/VI and UMEC/VI therapies. The lung dose and safety of all 3 agents, delivered from a single inhaler, are therefore expected to be similar to those in the approved FF/VI and UMEC/VI therapies. The doses, formulation, and inhaler investigated for the closed triple therapy have been shown to be suitable for progression into clinical COPD studies, allowing three drugs to be delivered simultaneously and offering the potential for improved compliance and outcomes in patients with advanced COPD. 

## Acknowledgments 

These two studies were funded by GSK (GSK study numbers: CTT116415 and 200587; clinicaltrials.gov registration numbers: NCT01691547 and NCT01894386, respectively). Programming support was provided for each study by GSK Bangalore. The authors would like to thank Emma Lindo for her statistical analysis assistance during the development of this manuscript. Editorial support in the form of development of the draft outline and manuscript first draft in consultation with the authors, editorial suggestions on draft versions of this paper, assembling tables and figures, collating author comments, copyediting, fact checking, referencing, and graphic services was provided by Ian Grieve, PhD and Katherine St. John, PhD at Gardiner-Caldwell Communications (Macclesfield, UK) and was funded by GSK. 

## Statement of interest 

Noushin Brealey, Alex Henderson, Rashmi Mehta, Ashutosh Gupta, and Jessica Renaux are employees of and hold stock in GSK. Ann Allen was an employee of and held stock in GSK at the time of study conduct and manuscript writing. 

## Supplementary information 

### Pharmacodynamic analysis (study 1)

Each derived endpoint was assessed using a mixed-effect model that included treatment and period as fixed effects, period-level and subject-level baseline as continuous covariates, and subject as a random effect. 

### Statistical analyses: sample size assumptions (studies 1 and 2)

The sample size calculation for both studies was based on the largest available within-subject standard deviation (SD) for C_max_ and AUC across the three components (FF, UMEC, VI) from previous studies. The largest available within-subject SD was 0.33 for study 1 and 0.34 for study 2 (based on study 1 data). 

Assuming a target sample size of 36, precision for the comparison of interest was estimated to be within 14% of the observed point estimate for both studies. Calculations were based on a symmetric two-tailed test procedure on the log_e_-scale and a type I error rate of 10%. Precision is expressed as the half-width of the 90% CI, and the lower and upper bounds of the 90% CI for the ratio would be within 14% of the observed ratio. 

Although the studies were an estimation-based approach, and no formal statistical hypotheses were to be tested, a sample size of 36 would provide sufficient power to use the bioequivalence criteria 0.80 – 1.25 as a benchmark when interpreting the results. 

### Analysis of VI pharmacokinetic parameters (studies 1 and 2)

In order to support the development of VI as a monotherapy, a third posthoc analysis investigated VI systemic exposure across treatment groups for the dual therapies FF/VI 400/100 μg vs. UMEC/VI 500/100 μg in studies 1 and 2. The concentration-time data were analyzed by noncompartmental methods. C_max_ and either AUC_(0–2)_ (study 1) or AUC_(0–6)_ (study 2) were analyzed separately using a mixed-effects model, with period and treatment as fixed effects and subject as a random effect. 

There was no difference in VI systemic exposure (C_max_ and either AUC_(0–2)_ or AUC_(0–6)_, for studies 1 and 2, respectively) for the FF/VI 400/100 and UMEC/VI(500)/100, and FF/VI 400/100 and UMEC/VI(250)/100 dual therapies in studies 1 and 2 (Supplementary Table 3).

## 

**Table 1.** Participant characteristics and demographics.

	Study 1 CTT116415 (n = 44)	Study 2 200587 (n = 44)
Mean age, years (range)	36.5 (20 – 56)	39.1 (19 – 66)
Sex, n (%) Male Female	32 (73) 12 (27)	36 (82) 5 (11)
BMI, kg/m^2^ (range)	27.7 (21.1 – 33.3)	N/A
Ethnicity, n (%) Hispanic or Latino Other	4 (9) 40 (91)	8 (18) 39 (89)
Race, n (%) African American/African heritage American Indian/Alaskan Native Native Hawaiian/Other Pacific Islander White (Arabic/North African heritage) White (Caucasian/European heritage)	38 (86) 1 (2) 1 (2) 0 4 (9)	34 (77) 1 (2) 0 1 (2) 8 (18)

BMI = body mass index.

**Table 2a.** FF Plasma PK summary in study 1 (CTT116415).

Parameter	Treatment^a^	N	n	Geometric mean (95% CI)	%CVb
AUC_(0–8)_ (h×pg/mL)	FF/UMEC/VI	43	43	383 (357 – 410)	23.0
	FF/UMEC	43	42	366 (333 – 402)	30.6
	FF/VI	42	42	322 (298 – 348)	25.5
AUC_(0–t)_ (h×pg/mL)	FF/UMEC/VI	43	43	882 (774 – 1,005)	44.3
	FF/UMEC	43	42	765 (653 – 897)	54.4
	FF/VI	42	42	662 (570 – 768)	50.7
C_max_ (pg/mL)	FF/UMEC/VI	43	43	79.4 (71.7 – 88.0)	34.2
	FF/UMEC	43	42	79.2 (71.0 – 88.3)	36.2
	FF/VI	42	42	63.6 (58.0 – 69.6)	29.9
t_1/2_ (h)^b^	FF/UMEC/VI	43	29	21.6 (17.2, 27.1)	65.0
	FF/UMEC	43	29	20.7 (16.7, 25.7)	62.2
	FF/VI	42	25	22.4 (17.6, 28.3)	62.5
t_max_ (h)^b^	FF/UMEC/VI	43	43	0.2 (0.1, 2.0)	NA
	FF/UMEC	43	42	0.5 (0.1, 2.0)	NA
	FF/VI	42	42	1.0 (0.1, 2.1)	NA
t_last_ (h)^b^	FF/UMEC/VI	43	43	36.0 (8.0, 48.2)	NA
	FF/UMEC	43	42	36.0 (8.0, 48.2)	NA
	FF/VI	42	42	24.0 (8.0, 48.1)	NA

^a^Total dose as 4 consecutive doses: 400 μg FF, 500 μg UMEC, and 100 μg VI. ^b^Median (min, max). AUC = area under the plasma concentration-time curve; C_max_ = maximum observed plasma concentration; CVb = between subject coefficient of variation; FF = fluticasone furoate; N = total number of subjects who received this study medication, n = number of subjects for whom parameter derived; NA = not applicable; PK = pharmacokinetics; t_1/2_ = terminal phase half-life; t_last_ = time of last measurable concentration; t_max_ = time to C_max_; UMEC = umeclidinium; VI = vilanterol.

**Table 2b.** FF plasma PK summary in study 2 (200587).

Parameter	Treatment^a^	N	n	Geometric mean (95% CI)	%CVb
AUC_(0–4)_ (h×pg/mL)	FF/UMEC(500)/VI	44	44	212 (196 – 229)	26.0
	FF/UMEC(250)/VI	43	43	211 (192 – 232)	31.8
	FF/VI	43	43	219 (200 – 239)	29.6
AUC_(0–t)_ (h×pg/mL)	FF/UMEC(500)/VI	44	44	629 (555 – 713)	43.2
	FF/UMEC(250)/VI	43	43	607 (525 – 702)	49.9
	FF/VI	43	43	644 (566 – 732)	43.8
C_max_ (pg/mL)	FF/UMEC(500)/VI	44	44	81.4 (73.3 – 90.6)	35.9
	FF/UMEC(250)/VI	43	43	81.1 (72.2 – 91.0)	39.1
	FF/VI	43	43	85.7 (76.6 – 95.8)	37.6
t_max_ (h)^b^	FF/UMEC(500)/VI	44	44	0.2 (< 0.1, 2.0)	NA
	FF/UMEC(250)/VI	43	43	0.2 (< 0.1, 2.0)	NA
	FF/VI	43	43	0.2 (< 0.1, 2.0)	NA
t_last_ (h)^b^	FF/UMEC(500)/VI	44	44	24.0 (4.0, 24.1)	NA
	FF/UMEC(250)/VI	43	43	24.0 (8.0, 24.1)	NA
	FF/VI	43	43	24.0 (8.0, 24.1)	NA

^a^Total dose as 4 consecutive doses: 400 μg FF, 500 μg or 250 μg UMEC, and 100 μg VI. ^b^Median (min, max).

**Table 3a.** UMEC plasma PK summary in study 1 (CTT116415).

Parameter	Treatment^a^	N	n	Geometric mean (95% CI)	%CVb
AUC_(0–4)_ (h×pg/mL)	FF/UMEC/VI	43	43	494 (459 – 531)	23.9
	FF/UMEC	43	42	498 (461 – 537)	24.9
	UMEC/VI	41	41	502 (463 – 544)	26.1
AUC_(0–t)_ (h×pg/mL)	FF/UMEC/VI	43	43	885 (785 – 997)	40.3
	FF/UMEC	43	42	797 (712 – 893)	37.6
	UMEC/VI	41	41	931 (810 – 1,070)	46.2
C_max_ (pg/mL)	FF/UMEC/VI	43	43	1,189 (1,015 – 1,392)	54.9
	FF/UMEC	43	42	1,099 (950 – 1,271)	49.2
	UMEC/VI	41	41	1,217 (1,016 – 1,459)	62.4
t_1/2_ (h)^b^	FF/UMEC/VI	43	12	24.6 (12.0, 50.4)	161
	FF/UMEC	43	19	14.3 (8.0, 25.4)	179
	UMEC/VI	41	15	17.8 (8.5, 37.4)	224
t_max_ (h)^b^	FF/UMEC/VI	43	43	0.1 (0.1, 0.2)	NA
	FF/UMEC	43	42	0.1 (0.1, 0.2)	NA
	UMEC/VI	41	41	0.1 (0.1, 0.3)	NA
t_last_ (h)^b^	FF/UMEC/VI	43	43	36.0 (8.0, 48.1)	NA
	FF/UMEC	43	42	24.0 (4.0, 48.2)	NA
	UMEC/VI	41	41	36.0 (6.0, 48.3)	NA

^a^Total dose as 4 consecutive doses: 400 μg FF, 500 μg UMEC, and 100 μg VI. ^b^Median (min, max).

**Table 3b.** UMEC plasma PK summary in study 2 (200587).

Parameter	Treatment^a^	N	n	Geometric mean (95% CI)	%CVb
AUC_(0–2)_ (h×pg/mL)	FF/UMEC(500)/VI	44	44	423 (383 – 467)	33.3
	FF/UMEC(250)/VI	43	43	205 (186 – 225)	31.6
	UMEC(250)/VI	43	43	204 (186 – 222)	29.3
AUC_(0–t)_ (h×pg/mL)	FF/UMEC(500)/VI	44	44	770 (688 – 862)	38.4
	FF/UMEC(250)/VI	43	43	323 (282 – 369)	45.6
	UMEC(250)/VI	43	43	310 (275 – 349)	40.4
C_max_ (pg/mL)	FF/UMEC(500)/VI	44	44	1,098 (910 – 1,326)	68.3
	FF/UMEC(250)/VI	43	43	540 (443 – 657)	71.1
	UMEC(250)/VI	43	43	549 (463 – 651)	59.9
t_1/2_ (h)^b^	FF/UMEC(500)/VI	44	15	4.1 (2.7, 6.2)	87.5
	FF/UMEC(250)/VI	43	22	2.3 (2.0, 2.6)	31.6
	UMEC(250)/VI	43	29	2.3 (2.1, 2.6)	27.4
t_max_ (h)^b^	FF/UMEC(500)/VI	44	44	< 0.1 (< 0.1, 0.2)	NA
	FF/UMEC(250)/VI	43	43	0.1 (< 0.1, 0.1)	NA
	UMEC(250)/VI	43	43	0.1 (< 0.1, 0.2)	NA

^a^Total dose as 4 consecutive doses: 400 μg FF, 500 μg or 250 μg UMEC, and 100 μg VI. ^b^Median (min, max).

**Table 4a.** VI plasma PK summary in study 1 (CTT116415).

Parameter	Treatment^a^	N	n	Geometric mean (95% CI)	%CVb
AUC_(0–2)_ (h×pg/mL)	FF/UMEC/VI	43	43	307 (289 – 327)	19.9
	FF/VI	42	42	260 (247 – 274)	17.1
	UMEC/VI	41	41	264 (247 – 282)	20.9
AUC_(0–t)_ (h×pg/mL)	FF/UMEC/VI	43	43	522 (482 – 565)	26.4
	FF/VI	42	42	462 (424 – 504)	28.5
	UMEC/VI	41	41	439 (401 – 481)	29.4
C_max_ (pg/mL)	FF/UMEC/VI	43	43	639 (586 – 696)	28.6
	FF/VI	42	42	442 (412 – 474)	22.8
	UMEC/VI	41	41	486 (445 – 514)	28.8
t_1/2_ (h)^b^	FF/UMEC/VI	43	39	8.6 (7.4, 10.0)	49.7
	FF/VI	42	38	9.0 (7.4, 10.9)	63.8
	UMEC/VI	41	38	7.6 (6.4, 9.0)	53.9
t_max_ (h)^b^	FF/UMEC/VI	43	43	0.1 (0.1, 0.2)	NA
	FF/VI	42	42	0.1 (0.1, 0.3)	NA
	UMEC/VI	41	41	0.1 (0.1, 0.3)	NA
t_last_ (h)^b^	FF/UMEC/VI	43	43	12.0 (4.0, 24.1)	NA
	FF/VI	42	42	12.0 (4.0, 24.2)	NA
	UMEC/VI	41	41	12.0 (2.0, 24.0)	NA

^a^Total dose as 4 consecutive doses: 400 μg FF, 500 μg UMEC, and 100 μg VI. ^b^Median (min, max).

**Table 4b.** VI plasma PK summary in study 2 (200587).

Parameter	Treatment^a^	N	n	Geometric mean (95% CI)	%CVb
AUC_(0–6)_ (h×pg/mL)	FF/UMEC(500)/VI	44	44	423 (402 – 445)	17.0
	FF/UMEC(250)/VI	43	43	403 (379 – 428)	20.0
	FF/VI	43	43	408 (384 – 433)	19.8
	UMEC(250)/VI	43	43	371 (347 – 396)	21.9
AUC_(0–t)_ (h×pg/mL)	FF/UMEC(500)/VI	44	44	498 (449 – 464)	23.4
	FF/UMEC(250)/VI	43	43	488 (398 – 467)	27.5
	FF/VI	43	43	507 (531 – 534)	27.3
	UMEC(250)/VI	43	43	436 (478 – 551)	30.5
C_max_ (pg/mL)	FF/UMEC(500)/VI	44	44	696 (580 – 646)	24.7
	FF/UMEC(250)/VI	43	43	638 (482 – 549)	31.4
	FF/VI	43	43	601 (701 – 749)	30.1
	UMEC(250)/VI	43	43	529 (581 – 658)	30.9
t_1/2_ (h)^b^	FF/UMEC(500)/VI	44	19	4.5 (3.5, 5.7)	53.0
	FF/UMEC(250)/VI	43	20	4.8 (3.6, 6.4)	65.9
	FF/VI	43	18	4.9 (4.0, 5.9)	39.7
	UMEC(250)/VI	43	19	4.7 (3.6, 6.2)	61.9
t_max_ (h)^b^	FF/UMEC(500)/VI	44	44	0.1 (0.1, 0.2)	NA
	FF/UMEC(250)/VI	43	43	0.1 (0.1, 0.2)	NA
	FF/VI	43	43	0.1 (0.1, 0.2)	NA
	UMEC(250)/VI	43	43	0.1 (0.1, 0.2)	NA

^a^Total dose as 4 consecutive doses: 400 μg FF, 500 μg or 250 μg UMEC, and 100 μg VI. ^b^Median (min, max).

**Table 5a.** Plasma PK comparisons from study 1 (CTT116415).

Component measured	Parameter	Treatment comparison	Treatment ratio (SE Log_e_) [90% CI for ratio]
FF	AUC_(0–8)_ (h×pg/mL)	FF/UMEC/VI vs. FF/UMEC	1.05 (0.029) [1.00 – 1.10]
		FF/UMEC/VI vs. FF/VI	1.17 (0.029) [1.12 – 1.23]
	C_max_ (pg/mL)	FF/UMEC/VI vs. FF/UMEC	1.01 (0.045) [0.94 – 1.09]
		FF/UMEC/VI vs. FF/VI	1.23 (0.045) [1.14 – 1.33]
UMEC	AUC_(0–4)_ (h×pg/mL)	FF/UMEC/VI vs. FF/UMEC	0.99 (0.032) [0.94 – 1.04]
		FF/UMEC/VI vs. UMEC/VI	0.98 (0.032) [0.93 – 1.04]
	C_max_ (pg/mL)	FF/UMEC/VI vs. FF/UMEC	1.09 (0.075) [0.97 – 1.24]
		FF/UMEC/VI vs. FUMEC/VI	0.99 (0.075) [0.87 – 1.12]
VI	AUC_(0–2)_ (h×pg/mL)	FF/UMEC/VI vs. FF/VI	1.20 (0.023) [1.15 – 1.24]
		FF/UMEC/VI vs. UMEC/VI	1.18 (0.023) [1.13 – 1.22]
	C_max_ (pg/mL)	FF/UMEC/VI vs. FF/VI	1.46 (0.037) [1.38 – 1.56]
		FF/UMEC/VI vs. UMEC/VI	1.33 (0.037) [1.25 – 1.42]

SE = standard error.

**Table 5b.** Plasma PK comparisons from study 2 (200587).

Component measured	Parameter	Treatment comparison	Treatment ratio (SE Log_e_) [90% CI for ratio]^a^
FF	AUC_(0–4)_ (h×pg/mL)	FF/UMEC(500)/VI vs. FF/UMEC(250)/VI	1.00 (0.032) [0.95 – 1.06]
		FF/UMEC(250)/VI vs. FF/VI	0.97 (0.032) [0.92 – 1.02]
	C_max_ (pg/mL)	FF/UMEC(500)/VI vs. FF/UMEC(250)/VI	1.01 (0.049) [0.93 – 1.09]
		FF/UMEC(250)/VI vs. FF/VI	0.95 (0.049) [0.87 – 1.03]
UMEC	AUC_(0–2)_ (h×pg/mL)	FF/UMEC(500)/VI vs. FF/UMEC(250)/VI	1.04 (0.031) [0.99 – 1.10]
		FF/UMEC(250)/VI vs. UMEC(250)/VI	1.00 (0.031) [0.95 – 1.06]
	C_max_ (pg/mL)	FF/UMEC(500)/VI vs. FF/UMEC(250)/VI	1.04 (0.048) [0.96 – 1.13]
		FF/UMEC(250)/VI vs. UMEC(250)/VI	0.98 (0.048) [0.91 – 1.07]
VI	AUC_(0–6)_ (h×pg/mL)	FF/UMEC(500)/VI vs. FF/UMEC(250)/VI	1.05 (0.024) [1.01 – 1.09]
		FF/UMEC(250)/VI vs. FF/VI	0.99 (0.024) [0.95 – 1.03]
		FF/UMEC(250)/VI vs. UMEC(250)/VI	1.09 (0.024) [1.04 – 1.13]
	C_max_ (pg/mL)	FF/UMEC(500)/VI vs. FF/UMEC(250)/VI	1.10 (0.032) [1.04 – 1.16]
		FF/UMEC(250)/VI vs. FF/VI	1.06 (0.032) [1.01 – 1.12]
		FF/UMEC(250)/VI vs. UMEC(250)/VI	1.21 (0.032) [1.14 – 1.27]

^a^Dose-normalized data.

**Table 6.** Pharmacodynamic assessments in study 1 (CTT116415).

Parameters	Change from baseline in weighted mean (0 – 4 hours)	Treatment comparison (test vs. reference)		
		FF/UMEC/VI vs. UMEC/VI	FF/UMEC/VI vs. FF/VI	FF/UMEC/VI vs. FF/UMEC
Maximum change from baseline in heart rate (bpm)	Test	18.9	18.9	18.9
	Reference	19.1	17.5	5.2
	Difference (95% CI)	–0.2 (–2.6 – 2.1)	1.4 (–0.9 – 3.7)	13.6 (11.3 – 15.9)
Derived heart rate (bpm)	Test	6.18	6.18	6.18
	Reference	6.08	5.97	0.69
	Difference (95% CI)	0.10 (–1.16 – 1.35)	0.20 (–1.05 – 1.46)	5.49 (4.25 – 6.73)
Minimum change from baseline in serum potassium (mmol/L)	Test	–0.30	–0.30	–0.30
	Reference	–0.37	–0.36	–0.24
	Difference (95% CI)	0.06 (–0.01 – 0.14)	0.05 (–0.02 – 0.13)	–0.06 (–0.13 – 0.01)
Derived serum potassium (mmol/L)	Test	–0.123	–0.123	–0.123
	Reference	–0.174	–0.185	–0.062
	Difference (95% CI)	0.051 (–0.022 – 0.124)	0.063 (–0.010 – 0.136)	–0.061 (–0.133 – 0.012)
Maximum change from baseline in blood glucose (mmol/L)	Test	0.544	0.544	0.544
	Reference	0.507	0.452	0.061
	Difference (95% CI)	0.037 (–0.044 – 0.110)	0.092 (0.011 – 0.172)	0.482 (0.403 – 0.562)
Derived blood glucose (mmol/L)	Test	–0.0211	–0.0211	–0.0211
	Reference	–0.0478	–0.0294	–0.1698
	Difference (95% CI)	0.0267 (–0.0354 – 0.0889)	0.0083 (–0.0539 – 0.0705)	0.1486 (0.0871 – 0.2102)

**Table 7.** Safety results for studies 1 (CTT116415) and 2 (200587)^a^.

Study 1	FF/UMEC/VI (n = 43)	UMEC/VI (n = 41)	FF/VI (n = 42)	FF/UMEC (n = 43)	Total (n = 44)
Any AEs, n (%)	4 (9)	6 (15)	6 (14)	3 (7)^b^	16 (36)
Study 2	FF/UMEC(500)/VI (n = 44)	FF/UMEC(250)/VI (n = 43)	FF/VI (n = 43)	UMEC(250)/VI (n = 43)	Total (n = 44)
Any AEs, n (%)	2 (5)	3 (7)	1 (2)	0	6 (14)

^a^Subjects who experienced at least 1 AE. ^b^In 1 subject, 1 of these AEs was identified as SAE (type 2 diabetes mellitus) resulting in the subject being withdrawn from the study. AE = adverse event; SAE = serious adverse event.

**Supplementary Table 1.** Summary of VI C_max_ plasma PK parameters from posthoc population PK modeling (study 1).

Input time (minutes)	Treatment^a^	N	Geometric mean, pg/mL (95% CI)
2	FF/UMEC/VI FF/VI UMEC/VI	43 42 41	614 (455 – 785) 616 (474 – 764) 620 (479 – 764)
3	FF/UMEC/VI FF/VI UMEC/VI	43 42 41	604 (457 – 780) 606 (464 – 767) 610 (464 – 768)
4	FF/UMEC/VI FF/VI UMEC/VI	43 42 41	599 (439 – 774) 600 (447 – 762) 605 (447 – 764)
5	FF/UMEC/VI FF/VI UMEC/VI	43 42 41	616 (489 – 775) 617 (502 – 722) 620 (508 – 740)
6	FF/UMEC/VI FF/VI UMEC/VI	43 42 41	631 (494 – 775) 634 (506 – 756) 637 (513 – 777)
7	FF/UMEC/VI FF/VI UMEC/VI	43 42 41	667 (514 – 858) 670 (520 – 862) 674 (529 – 866)
8	FF/UMEC/VI FF/VI UMEC/VI	43 42 41	686 (525 – 865) 689 (543 – 867) 693 (547 – 869)
9	FF/UMEC/VI FF/VI UMEC/VI	43 42 41	678 (541 – 809) 682 (558 – 810) 685 (558 – 810)
10	FF/UMEC/VI FF/VI UMEC/VI	43 42 41	669 (528 – 796) 671 (543 – 783) 675 (544 – 783)
11	FF/UMEC/VI FF/VI UMEC/VI	43 42 41	657 (514 – 777) 659 (528 – 767) 662 (543 – 768)
12	FF/UMEC/VI FF/VI UMEC/VI	43 42 41	652 (514 – 766) 655 (529 – 767) 657 (540 – 757)

^a^Total dose as 4 consecutive doses: 400 μg FF, 500 μg UMEC, and 100 μg VI.

**Supplementary Table 2.** Comparison of C_max_ across study 1, study 2, and the exploratory analyses of study 2.

Treatment	Treatment comparison^a^	Treatment ratio (90% CI for ratio)		
		Study 1 (CTT116415)	Study 2 (200587) original analysis	Study 2 (200587) exploratory analysis
FF	FF/UMEC(500)/VI vs. FF/UMEC(250)/VI	ND	1.006 (0.927 – 1.092)	0.967 (0.889 – 1.052)
	FF/UMEC(500)/VI vs. FF/VI	1.233 (1.144 – 1.328)	0.952 (0.877 – 1.033)	0.909 (0.836 – 0.989)
	FF/UMEC(250)/VI vs. FF/VI	ND	0.946 (0.872 – 1.026)	0.940 (0.864 – 1.023)
UMEC	FF/UMEC(500)/VI vs. FF/UMEC(250)/VI	ND	1.040 (0.960 – 1.127)	1.014 (0.933 – 1.101)
	FF/UMEC(250)/VI vs. UMEC(250)/VI	ND	0.983 (0.908 – 1.066)	1.000 (0.920 – 1.086)
VI	FF/UMEC(500)/VI vs. FF/UMEC(250)/VI	ND	1.095 (1.038 – 1.155)	1.157 (1.077 – 1.243)
	FF/UMEC(500)/VI vs. FF/VI	1.463 (1.376 – 1.556)	1.162 (1.102 – 1.226)	1.287 (1.198 – 1.382)
	FF/UMEC(250)/VI vs. FF/VI	ND	1.061 (1.006 – 1.120)	1.112 (1.036 – 1.195)
	FF/UMEC(250)/VI vs. UMEC(250)/VI	ND	1.205 (1.142 – 1.271)	1.250 (1.164 – 1.343)

^a^Total dose as 4 consecutive doses: 400 μg FF, 500 μg or 250 μg UMEC, and 100 μg VI. ND = analysis not done (there is no comparison as the lower UMEC dose was not evaluated in study 1).

**Supplementary Table 3.** Dual comparison for VI (studies 1 and 2).

Study	Parameter	Treatment comparison^a^	N	Ratio (90% CI)	%CVw
1	AUC_(0–2)_ (h×pg/mL)	FF/VI vs UMEC(500)/VI	41	1.016 (0.997 – 1.056)	10.6
	C_max_ (pg/mL)	FF/VI vs UMEC(500)/VI	41	1.097 (1.031 – 1.166)	16.9
2	AUC_(0–6)_ (h×pg/mL)	FF/VI vs UMEC(250)/VI	43	0.910 (0.874 – 0.947)	11.2
	C_max_ (pg/mL)	FF/VI vs UMEC(250)/VI	43	0.881 (0.835 – 0.930)	15.1

^a^Total dose as 4 consecutive doses: 400 μg FF, 500 μg or 250 μg UMEC, and 100 μg VI. CVw = within subject coefficient of variation.
